# Case Report: Occlusion of the foramen of Monro treated with endoscopic septostomy and foraminotomy in a preterm neonate

**DOI:** 10.3389/fsurg.2023.1257679

**Published:** 2023-12-15

**Authors:** Po-Yu Lai, Cheng-Hao Li, Ming-Tao Yang, Pao-Hui Feng, Chi-Man Kuok

**Affiliations:** ^1^ Division of Neurosurgery Department of Surgery, Far Eastern Memorial Hospital, New Taipei, Taiwan; ^2^ Department of Pediatrics, Far Eastern Memorial Hospital, New Taipei, Taiwan; ^3^ Department of Chemical Engineering and Materials Science, Yuan Ze University, Taoyuan, Taiwan

**Keywords:** foramen of Monro, septostomy, foraminotomy, sonography, endoscope, hydrocephalus

## Abstract

**Background:**

Hydrocephalus is a brain abnormality frequently encountered in neonates. There are several known etiologies of hydrocephalus, which can be classified as either congenital or acquired. Occlusion of the foramen of Monro (FOM) is a cause of hydrocephalus and can be either congenital or secondary to various disorders. Idiopathic obstruction of the FOM presenting as prenatal ventriculomegaly is extremely rare but has been reported.

**Case presentation:**

We present the case of a preterm newborn with severe bilateral hydrocephalus due to FOM occlusion in the prenatal period. Although the neonate had normal head circumference and no clinical symptoms after birth, brain sonography revealed progressive ventriculomegaly. Further image revealed bilateral ventriculomegaly with normal-sized third and fourth ventricles. We suspected a complete obstruction of the right and a partial obstruction of the left of the FOM. The neonate underwent endoscopic septostomy and foraminotomy, resulting in improvement.

**Conclusion:**

We report a case of preterm FOM occlusion in the youngest and smallest neonate to date. Endoscopic septostomy and foraminotomy were performed in order to avoid ventriculoperitoneal shunt-related complications, highlighting the viability of endoscopic procedures which should be the primary treatment in preterm neonates with FOM occlusion.

## 1. Introduction

Hydrocephalus is a condition that arises from an imbalance of cerebrospinal fluid (CSF) dynamics, which induces a pathological accumulation of CSF. This leads to an abnormal expansion of the cerebral ventricles and an increase in intracranial pressure. It is one of the most commonly observed abnormalities in the neonatal brain (1). Ultrasound is the primary imaging modality for evaluating neonatal brain abnormalities, while magnetic resonance imaging (MRI) has been shown to identify additional anomalies in approximately 20%–50% of cases detected by ultrasound (1).

Several etiologies of hydrocephalus have been reported, classified as either congenital or acquired (2). Post-hemorrhagic hydrocephalus of prematurity and congenital anomalies are the most common causes of hydrocephalus in high-income countries, whereas neonatal infections are predominant in developing countries (2). Infants with neonatal hydrocephalus may exhibit an abnormal increase in head circumference, irritability, vomiting, bulging of the anterior fontanelle, or splaying of the cranial sutures (2).

Occlusion of the foramen of Monro (FOM) is a cause of hydrocephalus and may be congenital or secondary to various disorders (3). Intraventricular and choroid plexus tumors can obstruct CSF flow, whereas vascular malformations and infectious etiologies can cause FOM obstruction due to mass effect or scarring (3). However, idiopathic occlusion of the FOM presenting as prenatal ventriculomegaly is extremely rare. In this article, we present a case of a newborn with bilateral hydrocephalus diagnosed during the prenatal period due to congenital and idiopathic occlusion of the FOM. The neonate underwent successful endoscopic septostomy and foraminotomy. To the best of our knowledge, this is the youngest and smallest neonate to have undergone this procedure.

## 2. Case presentation

### 2.1. Prenatal diagnosis and antenatal management

A 45-year-old woman was referred to our institution at 31 weeks of gestation with a suspected fetal brain anomaly. She had irregular prenatal examinations until 28 weeks of gestation, and a history of chronic hypertension with poor medical compliance was noted prior to pregnancy. Additionally, a high risk of preeclampsia was identified during this pregnancy. Third-trimester ultrasonography showed severe bilateral hydrocephalus (more severe on the right side) in the fetus.

The woman underwent emergency cesarean delivery at 31 weeks and 4 days due to severe maternal hypertension and fetal distress. A female newborn weighing 1,610 g (25–50th percentile) was delivered, with Apgar scores of 4 and 8 at 1 and 5 min, respectively. The maternal postoperative course was uncomplicated, and she was discharged at 4 days postpartum.

### 2.2. Postnatal management

**Figure 1:**
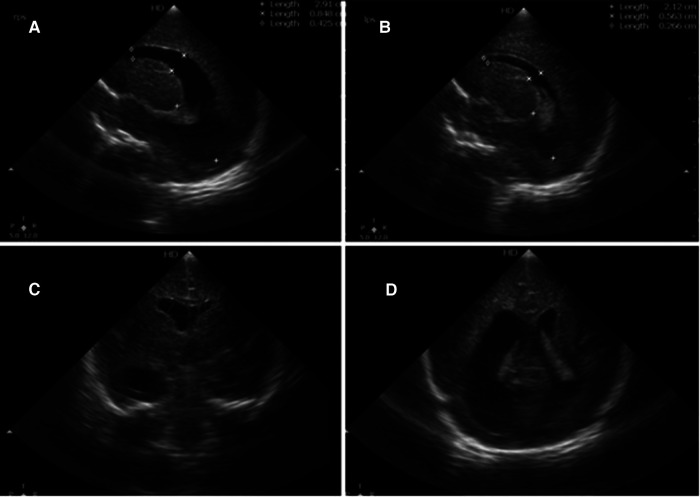
Brain sonography. (**A**) Right sagittal view, (**B**) left sagittal view, and (**C**,**D**) the coronal plane, showing ventriculomegaly, especially at the right side (**A**).

**Figure 2:**
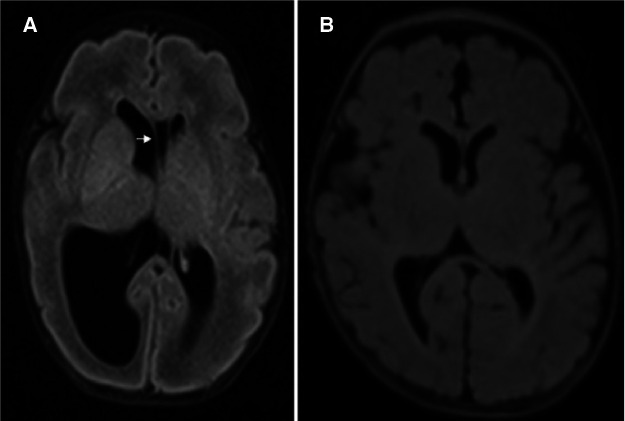
Preoperative T2 fluid-attenuated inversion recovery (FLAIR) sequence in the transverse view (**A**) showing bilateral ventriculomegaly and contralateral shift of the septum pellucidum (arrow), and postoperative T2 FLAIR sequence transverse view (**B**) showing regressive change of right-sided ventriculomegaly.

The neonate had an occipital-frontal circumference (OFC) of 29 cm (25–50th percentile), and a physical examination showed a flat anterior fontanelle. Brain sonography on the first day revealed bilateral ventriculomegaly, particularly on the right side, with midline deviation to the left side (Figure 1). The anterior horn width (AHW) of the lateral ventricle was 6.1 mm on the right side (97th percentile) and 3.2 mm on the left side (50–97th percentile). The thalamo-occipital distance (TOD) of the lateral ventricle was 29 mm on the right side (greater than 97th percentile + 6 mm) and 21 mm on the left side (greater than 97th percentile). The resistance index was normal, and the OFC remained stable. The neonate did not experience any clinical seizures in the following days. However, follow-up brain sonography every two days showed an increase in the lateral ventricle size. A neonatal brain MRI on the fifth day confirmed bilateral ventriculomegaly with the right side more severely affected than the left side. The MRI also revealed a left shift of the septum pellucidum (Figure 2A) and normal-sized third and fourth ventricles, which suggested a complete obstruction of the right and partial obstruction of the left of the FOM.

**Figure 3:**
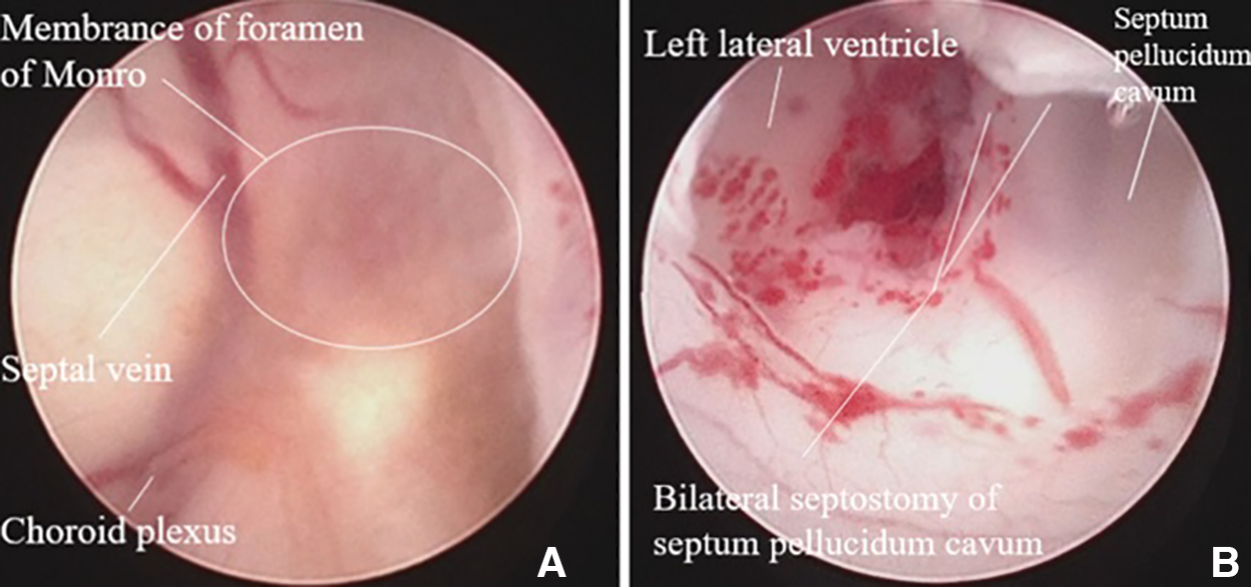
Intraoperative image showing obstructed foramen of monro, which was fenestrated to connect the bilateral ventricles.

Subsequently, at 8 days of life, the infant underwent neuroendoscopic approach, specifically a ventriculoscopy via the right Kocher's approach. The two layers of the septum pellucidum were fenestrated to connect the lateral ventricles bilaterally. The obstructed right FOM was identified by tracing the choroid plexus, and fenestration of the membranous obstruction of the FOM was conducted with membrane perforator and Fogarty catheter (Figure 3). To relieve the right lateral ventricle hydrocephalus and prevent left-sided hydrocephalus due to suspected partial obstruction, a Monro foraminotomy and septostomy were performed on the right side. The procedures were uneventful, and on the eighth postoperative day, brain sonography revealed a reduction in the size of the bilateral ventricles. Chromosomal abnormalities associated with ventriculomegaly were ruled out by karyotyping. Other potential causes, including congenital infections, were also negative. The infant was initially treated with nasal-prongs continuous positive airway pressure to relieve preterm respiratory distress and was successfully weaned off the ventilator. The infant was also able to tolerate the feeding program appropriately.

The patient was discharged on post operative day 52. Follow-up brain MRI performed 3 months after the operation showed significant regression of bilateral ventriculomegaly (Figure 2B). Brainstem auditory-evoked potentials and visual-evoked potentials were normal during outpatient follow-up. Developmental milestones were achieved by the age of 7 months (Supplementary Figure S1).

## 3. Discussion

Hydrocephalus has many etiologies, including neoplastic, congenital, inflammatory, infectious, and vascular malformations, which can lead to obstruction of the FOM (4). Among these, congenital and idiopathic obstructions of the FOM are rare, and symptoms may present either in childhood or adulthood (5). Common neurological symptoms in infants and children include seizures, bulging of the anterior fontanelle, abnormal head circumference, and delayed development (5). Congenital obstruction of the FOM during the prenatal period is extremely rare, and according to previous case reports (Table 1), not all cases present initially with an inappropriate head circumference or seizures. Some patients would be considered asymptomatic if not for findings through imaging. Hence, neuroimaging plays a crucial role in the early diagnosis and regular follow-up of hydrocephalus (1). MRI is highly effective in detecting FOM stenosis and ruling out other causes of lateral ventricle dilatation, such as obstructive cysts, tumors, vascular lesions, or meningeal lesions (1). Additionally, cranial sonography is a non-invasive and important tool for routine monitoring of ventricle size and flow pattern in cases with abnormal head circumference as well as asymptomatic patients with stable head circumference.

**Table 1:** Summary of previously reported cases with prenatal diagnoses of congenital FOM occlusion.

Study	Age at diagnosis (weeks)	Age at delivery (weeks)	Weight at diagnosis (g)	Clinical presentation	Operation time	Management	Affected side	Outcome
Hartung et al. (6)	36	38	2,330	Multiple organ anomalies	No intervention	No intervention	Left	Stillbirth
Gaston et al. (7)	36	37	3,780	Decreased tone	5 weeks after birth	VP shunt	Left	Appropriate developmental milestones at 8 months
Nakamura et al. (8)	29	30	848	Decreased tone	7 weeks after birth	VP shunt	Right	Normal at 1 year follow up
Patten et al. (9)	3rd trimester	–	–	–	<3 months	VP shunt	Unilateral	Normal
Patten et al. (9)	3rd trimester	–	–	–	<3 months	VP shunt	Unilateral	Intractable seizure and severe development delay
Patten et al. (9)	3rd trimester	–	–	–	<3 months	VP shunt	Unilateral	Normal
Anderson et al. (10)	20	36	2,280	Recurrent seizure	14 days after birth	VP shunt	Right	Global developmental delay, and seizure under medication control
Tsao et al. (11)	28	40	3,406	Wide sagittal suture	–	No intervention	Right	Normal at 9 months
Koga et al. (12)	34	38	3,460		25 days after birth	VP shunt	Left	Normal at 12 months
Chudley et al. (13)	–	40	4,010	1 week after birth Enlarged head circumstance	1 week after birth	VP shunt	Bilateral	Delayed expressive and receptive language
Senat et al. (14)	21	31	1,900	–	–	–	Left	Terminated
Schulman et al. (15)	35	40	3,240	Generalized hypertonicity	5 days after birth	VP shunt	Left	Mild right-sided hypertonicity at 6 weeks, but became normal at 5 months
Schulman et al. (15)	–	36	2,250	Normal	Not mentioned	VP shunt	Left	Normal psychomotor development at 11 months
Durfee et al. (16)	30.9	–	–	Not mentioned	Not mentioned	VP shunt	Unilateral	Developmental delay
Spennato et al. (17)	31	34 + 4	2,650	Enlarged cranial sutures	2 days after birth	Endoscopic septum pellucidotomy and foraminoplasty	Right	–

There is no consensus regarding hydrocephalus treatment timing among neurosurgeons, neonatologists, and pediatric neurologists. The maximum ventricle size in post-hemorrhage dilation preterm infants is related to neurodevelopmental outcomes, according to a previous study (18). Intervention is based on sequential cranial sonography findings and clinical symptoms including increased in ventricle size, splayed sutures, and bulging fontanelles (18). Brain sonography parameters include the ventricular index (VI), AHW, and TOD, where a VI >97th percentile + 4 mm, AHW > 10 mm, or TOD > 25 mm require neurosurgical intervention and treatment. In our case, the patient had no clinical symptoms, but sonography showed abnormal findings that fulfilled the intervention criteria. Therefore, cranial sonography is a valuable tool which can aid in timely surgical interventions for cases of progressive ventriculomegaly.

Previously, ventriculoperitoneal (VP) shunting was the preferred surgical strategy to alleviate symptoms of FOM occlusion (19). However, this procedure can result in complications such as mechanical malfunction and infection. Pediatric patients who undergo VP shunts to treat hydrocephalus are particularly predisposed to complications (20), with a prevalence of 20%, with infants below 1 year of age being the most affected. One study showed that most patients with shunt failure required re-shunting before reaching 2 years of age (21). In the past, isolated septum pellucidotomy was not a routine procedure, although it was sometimes performed together with foraminoplasty or in addition to third ventriculostomy (19). In current practice, minimally invasive endoscopic procedures are the commonly used management procedures. Endoscopic treatment is known to have excellent outcomes in the treatment of idiopathic bilateral FOM occlusion in adults (22). For neonates, a 10-year study demonstrated that endoscopy is a feasible treatment of post-hemorrhage or post-infectious hydrocephalus (23), but it did not include data for FOM occlusion.

Endoscopic septostomy enables CSF circulation between the obstructed and opposing ventricle, allowing communication with the third ventricle to resolve the obstruction and avoiding the need for a VP shunt (24). The patency rate of the initial endoscopic septostomy is 53%, which increased to 81% after repeated septostomy, ensuring improvement in isolated ventricular hydrocephalus in a study involving children (4). In addition, multiple shunt revisions increase the risk and failure rate of septostomy (4). Only a few complications have been reported from endoscopic septostomy, including sterile meningitis, intraventricular hemorrhage, and dehiscence of the operative wound incision (4). Published reviews provided limited data regarding the overall complications of endoscopic surgery in neonate. The advantages of neuroendoscopic techniques include invasiveness and potentially lower risk. However, more clinical experience in needed.

In the case of neonates, the FOM is relatively small and the tissues are fragile. This adds to the technical demands of the surgery, requiring the use of specialized small endoscopes and surgical instruments to ensure accuracy and safety. The limitation of neuroscopic approach include restricted visualization and the small size of instruments, highlighting the requirement for advanced technical expertise in this procedure. The limitation of neuroscopic approach include restricted visualization and the small size of instruments, highlighting the requirement for advanced technical expertise in this procedure.

To the best of our knowledge, our patient is the youngest and smallest neonate compared to other cases involving endoscopic septum pellucidotomy and foraminoplasty for treatment of FOM occlusion (Table 1). The first neonate to receive successful endoscopic treatment for FOM was in 2021 (17). The initial presentation of the first case involves an enlarged cranial suture, which differs from our case where only an enlargement in ventricle size in cranial sonography. Both of these two 2 cases demonstrate successful endoscopic treatment with positive outcomes. Historically, a VP shunt was the predominant treatment for neonates with congenital occlusion of the FOM with hydrocephalus. To avoid shunt complications, endoscopic septum pellucidotomy and foraminoplasty should be considered as primary treatment methods in neonates. However, the success and safety of the surgery depend on the experience and expertise of the surgeon, as these procedures have their limitations and challenges.

## 4. Conclusions

In conclusion, endoscopic septostomy and foraminoplasty are valuable treatment options to be considered in cases of congenital occlusion of the FOM with hydrocephalus in neonates.

## Data Availability

The original contributions presented in the study are included in the article/Supplementary Material, further inquiries can be directed to the corresponding author.

## Supplementary material

SUPPLEMENTARY FIGURE S1

Timeline of diagnostic and therapeutic events in this case.
