# Supplementary figures and images for: Case Report: Occlusion of the foramen of Monro treated with endoscopic septostomy and foraminotomy in a preterm neonate
Source: Front Surg. 2023 Dec 15;10:1257679. doi: 10.3389/fsurg.2023.1257679 (PMC10754961; doi:10.3389/fsurg.2023.1257679)

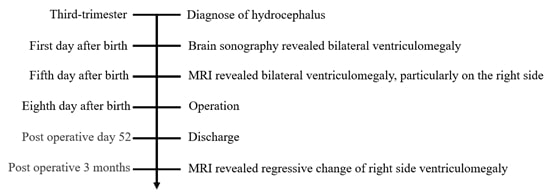

Supplement: Supplementary file 1 [file Image1.jpeg]
